# Hacking Aging: A Strategy to Use Big Data From Medical Studies to Extend Human Life

**DOI:** 10.3389/fgene.2018.00483

**Published:** 2018-10-23

**Authors:** Peter O. Fedichev

**Affiliations:** ^1^Gero LLC, Moscow, Russia; ^2^Moscow Institute of Physics and Technology, Moscow, Russia

**Keywords:** aging, mortality, biomarkers, healthspan, lifespan, longevity, biological age, frailty

## Abstract

Age is the most important single factor associated with chronic diseases and ultimately, death. The mortality rate in humans doubles approximately every eight years, as described by the Gompertz law of mortality. The incidence of specific diseases, such as cancer or stroke, also accelerates after the age of about 40 and doubles at a rate that mirrors the mortality-rate doubling time. It is therefore, entirely plausible to think that there is a single underlying process, the driving force behind the progressive reduction of the organism's health leading to the increased susceptibility to diseases and death; aging. There is, however, no fundamental law of nature requiring exponential morbidity and mortality risk trajectories. The acceleration of mortality is thus the most important characteristics of the aging process. It varies dramatically even among closely related mammalian species and hence appears to be a tunable phenotype. Here, we follow how big data from large human medical studies, and analytical approaches borrowed from physics of complex dynamic systems can help to reverse engineer the underlying biology behind Gompertz mortality law. With such an approach we hope to generate predictive models of aging for systematic discovery of biomarkers of aging followed by identification of novel therapeutic targets for future anti-aging interventions.

## 1. Introduction

Aging in most species, including humans, manifests itself as a progressive functional decline leading to the exponential increase in death risk from all causes. The mortality rate doubling time is approximately 8 years (Gompertz, 1825). Age-independent mortality mostly associated with violent death and infectious diseases has been progressively declining over the last century, mainly due to universal access to modern medicine and sanitation. The risks of death associated with the most prevalent age-related diseases remain very low at first, increase exponentially and dominate after the age of about 40 (Gavrilov and Gavrilova, 2005; Partridge et al., 2018). The incidence rates of the specific diseases, such as cancer or stroke, also accelerate after this age and double at a rate that closely tracks mortality acceleration (Barzilai and Rennert, 2012; Zenin et al., 2018). It is therefore, entirely plausible to think there is a single underlying driving force behind the progressive accumulation of health deficits, leading to the increased susceptibility to disease and death. This force is aging.

Although we have come to expect that physical decline is a natural consequence of aging, there is no natural law that dictates the exponential morbidity and mortality increase we observe among human populations. It is possible for death risks to increase very slowly, stay constant for extended periods, or even decline with age (Vaupel et al., 2004; Jones et al., 2014). Naked mole rats (Buffenstein, 2005; Ruby et al., 2018) and the growing number of bat species are now recognized as examples of mammals that exhibit the lack of detectable mortality acceleration, or negligible senescence (Finch, 1994). Formally, this means that the mortality rate doubling time could be arbitrarily large. In Kogan et al. (2015), we suggested that the mortality acceleration may vanish depending on the modifiable parameters, such as DNA repair or protein homeostasis maintenance efficiency (López-Otín et al., 2013), and should be, in principle, subject to manipulation. We propose to combine big data from large prospective observational studies with analytical tools borrowed from the physics of complex dynamic systems to “reverse engineer” the underlying biology behind the Gompertz law of mortality variables. This approach may yield mechanistic predictive models of aging for systematic discovery of biomarkers of aging, identification of novel therapeutic targets for future anti-aging therapies.

## 2. Human clinical data reveal a rich picture of aging trajectory

**Figure 1:**
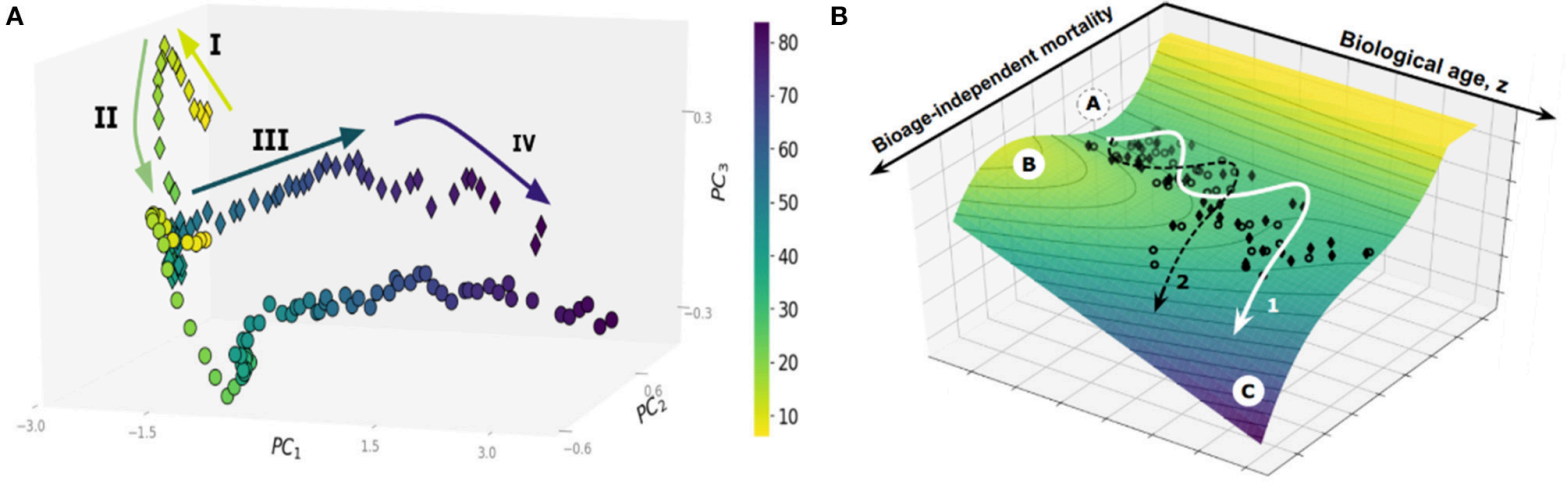
**(A)** Principal Component Analysis of human physical activity time series representations from an NHANES study. **(B)** The trajectory of aging is shown superimposed on the potential energy landscape (vertical axis), which provides a schematic visualization of the constraints provided by the underlying regulatory network. Each dot represents the physical activity state vectors of an age-and sex-matched cohort of NHANES participants (men, diamonds; women, circles). Cohorts were categorized by one year increments. The axes in the horizontal plane are (i) biological age (in years), and (ii) biological age-independent mortality. The stability basin A is separated from the unstable region C by the potential energy barrier B; The figures are adopted from Pyrkov et al. (2017).

Large cross-sectional datasets, such as the UK Biobank (UKB) or the National Health and Nutrition Examination Survey (NHANES), provide an invaluable window on the dynamics of human health as a function of age. Principal Component Analysis, a basic unsupervised learning technique especially useful for exploratory data analysis (Ringnér, 2008), reveals a sophisticated pattern of human development and aging, see Figure 1A. Each dot on the graph represents the averaged position of a person's organism state representations derived from one-week long physical activity tracks of NHANES participants, stratified into sex- and age-matched cohorts (Pyrkov et al., 2017). The data distribution paints a complex multidimensional picture beyond the obvious overall decline in physical activity levels in the sick and elderly. On the coarse-grained level, however, the life history appears as a well-defined trajectory in the physiological parameters space. State dynamics are distinctly different among age ranges corresponding to childhood (below, approximately 15 years old), young adult and adult stages (before and after the age of approximately 40 years old, respectively), followed by yet another distinct phase in old age marking at the end of the “healthspan.” Healthspan is defined (Fries, 2002) as the age at which the first debilitating disease appears, followed by multiple linked morbidities, frailty, and eventually death.

In the dynamics systems theory framework, the restriction of the variation in physiological variables to the low-dimensional aging trajectory has a deep physical significance. Biological systems consist of strongly interacting components built from an enormous number of individual parts and thus belong to the realm of statistical physics or physical kinetics. Under most common conditions, the state and the dynamics of such complex systems can be described by a very few “macroscopic” variables (Lifshitz and Pitaevskii, 1981; Pitaevskii and Lifshitz, 2012). The necessity for the correlation between the vital physiological variables over spatial and time scales, representing the organism's size and lifespan, as well as evolutionary pressure, drives the underlying regulatory networks to criticality (Hidalgo et al., 2014; Krotov et al., 2014). The order parameter, associated with the unstable phase, is the emergent organism level property characterized by extensive relaxation time, amplified response to perturbations, and coinciding, approximately, with the first principal component score. This is, therefore, a natural biomarker of age, or the biological age, that can be approximately identified in any sufficiently large dataset by means of PCA. It is closely related to Strehler-Mildvan vitality (Strehler and Mildvan, 1960) deficit, a qualitative measure of deviations from the youthful state. The time scales involved with the biological age dynamics are long (compared to mortality rate doubling time) and naturally correspond to the life stages spanning development (Krotov et al., 2014) and aging (Podolskiy et al., 2015).

## 3. Aging trajectories and the biomarkers of age and frailty

The profound linear association of most physiologically relevant variables with age is a hallmark of aging studies in human subjects and, therefore, can be used to construct useful “biological clocks.” Typical biological age models involve linear regressions of physiologically relevant variables to chronological age. Examples of this include IgG glycosylation (Krištić et al., 2013), blood biochemical parameters (Levine, 2012; Putin et al., 2016), gut microbiota composition (Odamaki et al., 2016), and cerebrospinal fluid proteome (Baird et al., 2012). To date, the “epigenetic clock” based on DNA methylation (DNAm) levels (Hannum et al., 2013; Horvath, 2013) appears to be the most accurate measure of aging, showing remarkably high correlation with chronological age. The DNAm clock predicts all-cause mortality in later life better than chronological age (Marioni et al., 2015). The biological age acceleration (BAA) is defined as the difference between the biological age estimation of an individual and the average biological age prediction in the sex- and the age-matched cohort. This indicator is elevated in patients with chronic diseases, such as HIV (Zhang et al., 2016), Down syndrome (El Hajj et al., 2016), or obesity (Horvath et al., 2014).

The BAA predicts healthspan (Pyrkov et al., 2017) and therefore healthspan can be used as a simple BAA proxy. We produced a GWAS of healthspan and observed (Zenin et al., 2018) strong (|ρ_*g*_| > 0.3) genetic correlations between the healthspan and the risks of specific age-related disease (with the notable exception of dementia). Other examples of strong genetic correlations included traits such as all-cause mortality (as derived from parental survival, with ρ_*g*_ = −0.76), life-history traits (metrics of obesity, age at first birth), levels of different metabolites (lipids, amino acids, glycemic traits), and psychological traits (smoking behavior, cognitive performance, depressive symptoms, insomnia). It is therefore, plausible to think that chronic age-associated diseases share components of their genetic architecture, which further supports the hypothesis of shared underlying mechanisms. Future understanding of genetic factors of predispositions to chronic diseases and accelerated aging can help to improve the accuracy of Health Risk Assessment (HRA) in life insurance, personal wealth management, and retirement planning applications.

Popular biological age models are trained to predict chronological age, however, often fail to fully capture signatures of mortality and incidence of diseases. This deficiency can be addressed with log-linear risk models using, if available, the clinical or death registry to produce a biological age estimation in the form of log-hazard ratio (Liu et al., 2018; Pyrkov et al., 2018). In Pyrkov et al. (2017), we observed, however, that mortality prediction has a significant age-independent component associated with chronic diseases burden or clinical frailty index. The nature of the variation of physiological parameters associated with age (the “aging drift”) and its relation to chronic diseases and death is thus compatible with the following semi-quantitative picture, see Figure 1B (Pyrkov et al., 2017). The organism state dynamics in the highly multidimensional space of all possible biological measurements is constrained by an unstable effective potential defined by the underlying regulatory interactions. The organism state slowly drifts along the “soft” direction along the free energy basin associated with the least curvature. The systematic shifts and fluctuations on top of the aging drift represent the organism's responses to perturbations, such as diseases or lifestyles, such as smoking.

Survival depends on the shape of the potential barriers separating the healthy aging individuals from the dynamically unstable regions, see Figure 1B. As the organism state changes, the nature of the regulatory interactions also vary: it is natural to assume that there is at least one potential barrier, with the activation energy decreasing as a function of (biological) age. Accordingly, the Gompertz mortality law arises from the exponentially increasing chances of a stochastic activation over the lowest of the barriers and transiting into a relatively short-lived state characterized by the complete loss of dynamic stability, multiple morbidity, and death. In this picture, the increase in biological age is not an indicator of any specific disease. Instead, it drives the build-up of functional deficits, loss of resilience, and exponentially rising risks of incidence of chronic diseases.

## 4. An effective strategy to extend human lifespan

The form of the effective potential constraining the evolution of physiological state variables on the time scales relevant to aging and diseases broadly suggests that there could be two possible strategies for human life extension. One option would be to target resilience with interventions that increase the height of the barrier with the least activation energy at any given age without counter-acting the aging drift (see Figure 1B). To our knowledge, there are few examples pointing to such a possibility. It appears from the analysis that smoking does not affect the aging drift but instead, reduces the resilience, thus increasing the chances of disease and death (Pyrkov et al., 2017). The effect of smoking is reversible, with individuals who quit smoking before a certain age experiencing a similar life expectancy to their peers who have never smoked (Taylor et al., 2002). There is experimental evidence that caloric restriction in flies produces another example of reversible short-term death risk without appreciable changes in the rate of aging (Mair et al., 2003).

The other possibility would be to introduce a therapy aimed at the reduction of biological age itself. This option is considerably more attractive, since it would imply an action against the slowest mode causally involved in the loss of resilience and hence would produce a long-lasting effect on healthspan and survival. The intervention would mitigate health deficits, delay the onset of chronic diseases and henceforth bring substantial improvements in quality of life. A transient rapamycin treatment in mice leads to a significant life extension, changes the disease incidence statistics long after the cessation of the treatment (Bitto et al., 2016), and may, therefore, serve as an example to inspire such a true rejuvenation attempt.

## 5. Practical considerations

Using an example from manufacturing, a complicated machine in hand can be studied and reproduced, or “reverse engineered” with insights about its function gleaned through the study of its form. Reverse engineering is easier than invention from scratch, which is why advanced electronic devices or military machines are guarded secrets. Any proposal involving biological reverse engineering and subsequent targeting of the regulatory subsystem responsible for the control of the aging process necessarily implies data acquisition. Aging models are then inferred from the data to identify aging regulators or potential anti-aging therapies. The physical kinetics equations are signal-agnostic, and hence the choice of the specific biological variables for the analysis should be driven by additional requirements such as data quality, availability, and actionability. Other important factors include the ease of preclinical validation and the expected regulatory burden. Biological studies involving a large number of samples are costly and logistically involved. The criticality of the underlying regulatory network dynamics greatly facilitates the analysis, since it implies a separation of scales between aging dynamics and considerably faster reversible responses of the organism to specific stress factors. Therefore, it should be possible to obtain a sufficiently complete quantitative picture of the aging process, including the system of regulators of aging, in a cost-efficient way from a minimum number of samples representing aging organisms.

For example, the increasing number of available genomes of exceptionally old and hence successfully aging individuals can provide an insight on the genetic architecture of exceptional life- and health- spans by use of Genome-Wide Association Studies (GWAS). The genetic variants associated with extreme lifespan, including parental longevity (Joshi et al., 2016), or healthspan (Zenin et al., 2018) may serve to predict transcriptomic signatures of longevity or BAA. If combined with large drug perturbation databases, such as the Broad Institute CMAP, the results of genetic studies could be used for transcriptomic GWAS-imputation followed by ranking small molecular compounds as potential life-extending interventions, or drug repositioning (So et al., 2016). Mining transcriptomic signatures of drug perturbations to counter aging drift in gene expression levels has a long history of success in model organisms (see e.g., Tarkhov et al., 2018 for our recent example of identification of experimental drugs extending lifespan in nematodes). Redirecting existing drug therapies for new applications is particularly attractive since it potentially sidesteps the target ID, and validation steps (although many drugs are well-characterized, permitting a robust target hypothesis). Once the efficacy of the predicted drugs is confirmed in animal studies, FDA approval could be safely expedited for human clinical trials.

Some specific genetic variants from the GWAS could hint at attractive targets for future genetic therapies against aging. Alternatively, a sufficiently large dataset of gene expression in a cohort of aging human subjects may yield an entirely new set of targets for a genetic intervention, including RNA interference (Wittrup and Lieberman, 2015), gene editing (Cox et al., 2015), or over-expression of predicted genes using a viral vector for delivery (Naldini, 2015). All the technologies are in their first steps in clinical trials and medical applications and yet they could be selected for state-of-art anti-aging therapeutics. Compared with the development of small molecules against target proteins, the products of the selected genes, the approach could provide a valuable alternative to the small molecule drug discovery, by mitigating the uncertainty related to the difference in action on the gene transcript and gene product in complex cellular environments.

Another window of opportunities arises from the recent progress in the fields of targeted metabolomics and high-throughput proteomics combined with increasing availability of stored tissue samples from richly characterized patients. Investigations of aging dynamics and control of the circulating blood plasma metabolites and proteins is an especially exciting opportunity, since it is supported by experiments with young blood transfusion (Villeda et al., 2014) and parabiosis (Conboy and Rando, 2012). Early results from clinical trials suggest that human blood contains a plethora of biological signals responsible for intracellular communication and synchronization, including those associated with development and aging. It is, therefore, promising to use aging dynamics models to identify putative regulators of aging among circulating molecules. The novel metabolites could be patented in some jurisdictions to support development costs and could be used directly or as templates for novel therapeutics (Martens et al., 2018). The harmful proteins could be selectively removed from circulation by a medical device performing extracorporeal adsorption via therapeutic apheresis. Targeting both kinds of circulating targets offers a substantial reduction in the number and volume of necessary regulatory studies and the associated risks and costs of a successful proof of concept study in humans. The GWAS of longevity or healthspan can be very useful in conjunction with the longitudinal targeted proteomics or metabolomics analysis to provide extra-evidence for life-extending potential of the suggested targets.

Animal preclinical studies are required to prove the efficacy of any proposed therapeutic solution. Experiments with nematodes and fruit flies offer short turnaround times and may yield relevant information since many genetic pathways controlling aging turn out to be evolutionary conserved (Smith et al., 2008). Preclinical studies require experiments with mammals, such as mice. The lifespan of mice is relatively long (more than 100 weeks), thus prompting the inclusion of surrogate endpoints associated with lifespan and functional state of the organism, such as Physiological Frailty Index (Antoch et al., 2017), Frailty Indices (Rockwood et al., 2017), or DNA methylation age (Petkovich et al., 2017; Stubbs et al., 2017) measurements in aging animals in response to anti-aging interventions.

The biomarkers of age and frailty should, in principle, be detectable consistently in a variety of vital signs typically available from large datasets from human studies. It is therefore possible to choose any convenient subset of physiological variables based on costs, signal-to-noise ratio, or regulatory considerations. We proposed using human physical activity tracks (Pyrkov et al., 2017) for two reasons: first, the overall level of activity is positively associated with healthspan (see e.g., Demontis et al., 2013); second, the relevant data is routinely collected and stored online by ubiquitous wearable devices (including mobile phones) for hundreds of millions of individuals all over the world. We demonstrated that BAA estimations can be produced on a server and reported back to users via a mobile phone application. The accuracy of the biomarker can be further improved with the help of modern machine learning tools, such as deep convolution neural networks (Pyrkov et al., 2018).

It is not easy to introduce a novel biomarker of age into clinical practice. It is therefore, necessary to develop novel clinical trial designs and protocols involving measures of functional decline and reliable surrogate endpoints with the goal to control health deficits associated with “healthy aging” in otherwise healthy individuals as early as in mid-life. The much anticipated 11th Revision of the International Classification of Diseases (ICD-11) introduces a number of aging-related conditions such as age-associated cognitive decline (MB21.0). This is the first step for medical professionals and healthcare systems worldwide to identify novel pathways for the development of therapeutic interventions from regulatory and market access standpoints. This should facilitate new clinical trials and market authorization of therapies aimed at functional declines associated with aging.

Finally, the interventions against aging should be applied early in life and hence must be exceptionally safe to let the long-term benefit (reductions in disease and mortality risk) outweigh the risks of adverse events. Ideally, the therapies selected by their effect on aging should produce a lasting effect after a single or a short series of interventions. Such an ideal approach should lead to an accumulation of the benefits of subsequent treatments and minimize unwanted side-effects.

## 6. Conclusions

Big data from electronic medical records and research databases offer a whole new way to understand aging. The exponential increase of morbidity incidence and mortality rate, the hallmarks of aging, can finally be traced to the variations in physiological variables among individuals, jointly describing the organism state in response to the multitude of external stresses and conditions and endogenous factors controlling the development and aging.

The identification of biological age from the biomedical data could be a way to translate the most recent findings from fundamental aging research into life insurance first and then, eventually, to clinical and medical settings. We envision the joint use of personalized genomics and streamed wearable sensor data for continuous monitoring of patient's health and risks of diseases and death. In the future, one may think of an advanced AI system following life histories of millions of people and feeding back real-time recommendations to reduce biological age and improve resilience measures and prolong healthspan of subscribed individuals.

Given a substantial progress in establishing biomarkers of age, the research shifts to the inference of the relations between the molecular level variables, such as expression levels of individual targets, to long-term outcomes including the incidence of chronic diseases and death. This should open a way to the rational design of an entirely new class of therapeutics, aimed specifically at mitigating health deficits, improving resilience, and increasing healthspan.

## Author contributions

The author confirms being the sole contributor of this work and has approved it for publication.

### Conflict of interest statement

PF is a shareholder of Gero LLC.
